# Additional Investigations and Referrals by Primary Care Physicians in Uncertain Clinical Situations: Cross-Sectional Study Using Virtual Patient–Based Scenarios

**DOI:** 10.2196/71717

**Published:** 2026-03-30

**Authors:** Mathieu Lorenzo, Thierry Pelaccia, Emmanuel Triby, Yunus Can, Romain Krider, Hubert Maisonneuve, Nicolas Fernandez

**Affiliations:** 1 Department of General Medicine and Territorial Training, Faculty of Medicine, Midwifery, and Health Sciences, University of Strasbourg, 4 rue Kirschleger, Strasbourg, 67085, France, 33 03 68 85 34 55; 2 Center for Training and Research in Health Sciences Education (CFRPS), Faculty of Medicine, Midwifery, and Health Sciences, University of Strasbourg, Strasbourg, Grand Est, France; 3 Faculty of Education and Lifelong Learning (ESPE), University of Strasbourg, Strasbourg, France; 4 University Institute for Primary Care, Faculty of Medicine, University of Geneva, Geneva, Switzerland; 5 Center for Applied Pedagogy in Health Sciences (CPASS), University of Montreal, Montréal, QC, Canada

**Keywords:** clinical decision-making, uncertainty, patient simulation, primary care anxiety, diagnostic tests, referral and consultation

## Abstract

**Background:**

Uncertainty affects at least 20% of primary care consultations, possibly leading primary care physicians to order additional investigations or referrals, affecting cost-effectiveness and patient safety. Experience is a key determinant in making these orders, along with anxiety or physicians’ reactions to uncertainty. Past studies addressing the links between experience and additional investigations and referrals in uncertain situations have used questionnaires, database analyses, or interviews with general practitioners, but no study has used fully standardized conditions.

**Objective:**

This study aimed to examine the association between experience and orders for additional investigations and referrals in uncertain situations using a standardized virtual patient simulation.

**Methods:**

A cross-sectional study was conducted with 40 physicians stratified by sex and experience (<10 vs ≥10 years). Participants engaged in a simulated clinical scenario involving a man aged 69 years with atypical dyspnea designed to evoke diagnostic uncertainty. The virtual patient was presented via first-person video to assess the physicians’ decision-making process. Participants’ years of clinical experience, sex, age, place of practice, type of practice, number of in-office patients, duration of consultations, State-Trait Anxiety Inventory Form Y (STAI-Y) and Physician Reaction to Uncertainty scores, and diagnostic hypotheses were collected and analyzed using multivariate regression models.

**Results:**

The group with <10 years of experience had higher STAI-Y (mean 41.3, SD 6.8 vs mean 32.7, SD 8.2; *P*<.001) and Physician Reaction to Uncertainty (mean 20.7, SD 5.4 vs mean 14.4, SD 6.9; *P*=.002) scores. Participants with <10 years of professional experience ordered more additional investigations and referrals on average: 10.2 (SD 3.4; 95% CI 8.9-11.7) vs 8.1 (SD 3.7; 95% CI 6.5-9.9; *P*=.03). There was no effect on costs: €153.80 (US $173.45) vs €129.60 (US $146.16) (effect size 0.296, 95% CI −0.348 to 0.939; *P*=.23). Multivariate analysis showed an association between the number of additional investigations and referrals with age (relative risk 0.980, 95% CI 0.963‐0.997; adjusted *P*=.02) and mean STAI-Y score (relative risk 0.984, 95% CI 0.968‐0.999; adjusted *P*=.04) but not with experience (<10 vs ≥10 years; *R*^2^=0.308).

**Conclusions:**

Less experienced physicians do not appear to overly rely on additional investigations and referrals in uncertain situations, suggesting that clinical reasoning remains a well-preserved skill among the younger generation of physicians. Future research could explore interventions targeting physicians’ anxiety and tolerance to uncertainty as potential factors influencing requests for additional investigations and referrals. In practical terms, two avenues could be explored: specific training for supervisors to help them address uncertainty tolerance in the feedback they provide to trainees and the use of virtual consultations as a complement to traditional training, with particular attention to this aspect.

## Introduction

In primary care, at least 20% of consultations are in situations of uncertainty [1,2]. In such situations, primary care physicians (PCPs) sometimes order additional investigations, such as biological testing or radiological analyses, or offer to refer patients to specialists to help decision-making [1,3-6]. Optimal management of additional investigations and referrals is an objective for cost-effectiveness and patient safety [7-10]. Numerous papers suggest that younger generations of practitioners may be losing clinical skills and using too many additional investigations and referrals [11-13].

Experience appears to be a key determinant in additional investigations and referrals ordering [14-17]. In addition to experience, several factors have been singled out in the literature for their possible influence on additional investigations and referrals ordering in situations of uncertainty. Some relate to conditions of practice, such as the time available for each patient or the availability of tests or specialists [1,5,14,18,19], or the existence and provision of forms for requesting tests [20]. Others include societal factors, such as the fear of possible prosecution or the low cost of tests in some health care systems [5]; practitioners’ clinical reasoning [14,20-24]; practitioners’ emotional reaction to uncertainty, fear of regrets, and anxiety [5,14,19-21,23,25,26]; influence of the patient [1,5,14,26,27]; practitioners’ expectations of performance [5]; or practitioners’ sex [18]. Anxiety, in particular, had been shown to be correlated with additional investigations and referrals ordering in uncertainty [5,14,23].

In studies addressing the links between experience and additional investigations and referrals in situations of uncertainty, data were obtained using questionnaires [23], database analyses [18], or interviews with general practitioners [5,14,15,26]. To our knowledge, no study has been conducted under fully standardized conditions. Standardization involves adhering to 4 key principles: all students complete the same task, all receive identical and clear instructions, no student benefits from an undue advantage, and a predetermined grading system is applied consistently across all participants [28]. This approach ensures a fair and equitable evaluation process while minimizing the influence of external factors, such as patient variability, on additional investigations and referrals performance. Thus, to gain a better understanding of the links between experience and additional investigations and referrals in decision-making in situations of uncertainty, we believe that using a standardized situation, presented by virtual patients, is a sound approach.

The objective of this study was to explore the association between experience and additional investigations and referrals in uncertain situations, using virtual primary care consultations.

## Methods

### Measurement Tool

We have developed a computer tool (Hello-Clinical; University of Strasbourg) that makes it possible to simulate virtual consultations to assess the decision-making process of health care professionals for training or research purposes under standardized conditions. This study represented the first use of this tool.

We divided our virtual clinical consultations into 5 stages [4] to collect the participants’ diagnostic hypotheses at each stage in an open text field without suggestions for responses. The virtual patients were presented through first-person video recordings. At the beginning, the patient is seen standing in the waiting room and greeting the physician with “good morning” after being called by the PCP. The first set of diagnostic hypotheses is collected at this stage based on initial cues such as the patient’s appearance and apparent health status.

Next, the patient sits down at the PCP’s desk and explains the reason for the visit. A second set of diagnostic hypotheses is then collected.

The PCP can then interact with the patient by typing questions in a text field. An algorithm automatically selects and displays the appropriate video response. For example, if the PCP asks, “Have you had a fever since you started feeling tired?” a video is shown of the patient responding, “No, I haven’t noticed any fever recently.”

After another round of diagnostic hypothesis collection, the PCP may request parts of the clinical examination. For instance, if blood pressure is requested, the corresponding values appear on the screen. A final diagnostic hypothesis collection follows, after which the PCP can write the additional investigations and referrals deemed appropriate in an open text field.

### The Population Under Study

We recruited a sample of community-based PCPs practicing ambulatory medicine in the Grand Est region of France, including urban and rural settings. Physicians worked in nonacademic practices, either solo or group based.

The sampling strategy was predefined and stratified according to sex and professional experience. We aimed to include similar numbers of men and women to reflect the gender distribution of PCPs in the region, as well as comparable numbers of physicians with less than and more than 10 years of professional experience. The 10-year threshold was chosen based on previously published work on the development of expertise [29-33].

Recruitment followed a structured snowball procedure [34]: 2 initial PCPs known by ML were contacted and agreed to participate. Each participant was then asked to systematically provide the contact details of 1 to 3 additional PCPs. Potential participants were contacted via phone or email using a standardized information procedure. Oral informed consent was obtained prior to data collection.

Although the initial inclusion criterion was being an actively practicing PCP, recruitment was subsequently guided by ongoing monitoring of the stratification variables. Additional participants were specifically sought to meet predefined sex and experience quotas. Data collection took place between June 2017 and September 2017.

### Virtual Clinical Case

We opted to use a clinical virtual vignette to simulate a scenario in which even experienced general practitioners would likely consider requesting additional investigations and referrals. Specifically, we designed the vignette around an atypical case of pulmonary embolism. The script portrayed a 69-year-old man presenting with progressive dyspnea over several days. The clinical presentation was intentionally vague, with the goal of creating diagnostic uncertainty. The goal was to contextualize the participants’ decision-making by attempting to reproduce an authentic context. Details of the main characteristics of the virtual patient are provided in Textbox 1.

Participants were able to familiarize themselves with the software beforehand by tackling a tutorial script on a 60-year-old man with recent, unexplained asthenia.

Textbox 1. **Main characteristics of the virtual patient used in a cross-sectional study on experience and additional investigations and referrals requests by primary care physicians in uncertain situations using standardized virtual patient simulations in France in 2017.**

Medical history

- Smoking ceased in 2012: 20 pack-years
- High blood pressure since 2010
- Right ulnar head fracture in 1989
- Peritonitis in 1974

Biometrics in the virtual case

- 85 kg
- 1.86 m
- BMI 24.5 kg/m^2^
- Blood pressure 132/76 mm Hg
- Pulse 110 bpm

Treatment

- Hydrochlorothiazide 25 mg/day

### Variables Collected

We identified various variables known to influence additional investigations and referrals ordering in situations of uncertainty [1,5,14,15,18,20,21,23]: participants’ sex, age, years of clinical experience, anxiety, place of practice, type of practice, average number of in-office patients, and average duration of consultations. Experience was defined as the number of years of practice since graduation. Place of practice (urban, rural, or semiurban), type of practice (alone or association), number of regular in-office patients, and average duration of consultation were related to practice conditions [5,14,15,18]. These data were collected through a questionnaire submitted after the simulated consultations. No confounding patient-related factors were collected, given the use of a standardized patient. Test availability was set to maximum in this virtual clinical case. We measured the participants’ anxiety levels using the State-Trait Anxiety Inventory Form Y (STAI-Y) scale [35,36] in its French version, observing only the state-anxiety dimension to save time. We selected this instrument because it is a widely used and validated measure of anxiety, with a validated French version available [35,37]. It has been used in PCP trainees [38]. We gathered responses to the French version of the Physician Reaction to Uncertainty (PRU) questionnaire [39,40]. This questionnaire has been used previously in PCP populations [40,41]. We then asked participants to rate the uncertainty and authenticity of the virtual clinical case from 1 (no uncertainty) to 5 (complete uncertainty) to verify if the case was an uncertain situation for them.

ML, YC, and RK scored the severity of the final diagnostic hypotheses based on the French clinical classification of emergency patients (classification clinique des malades des urgences; CCMU):

- CCMU 1: clinically stable, no immediate risk
- CCMU 2: condition requiring prompt attention, but no immediate life-threatening risk
- CCMU 3: potentially serious condition with a risk to life or function, requiring urgent care
- CCMU 4: very serious condition with a short-term life-threatening prognosis
- CCMU 5: deceased

Additional investigations and referrals were collected after the final stage of diagnostic hypothesis generation, categorized, and assigned costs according to the French national health pricing system (Caisse Nationale de l’Assurance Maladie des Travailleurs Salariés; CNAMTS).

### Statistical Analysis

In the absence of literature on the subject, we made an empirical hypothesis that PCPs with less than 10 years of experience would order a mean number of additional investigations and referrals of 10 (SD 6.0) and PCPs with 10 years or more of experience would order a mean number of additional investigations and referrals of 5 (SD 2.5). We then had to include 20 PCPs in each group to obtain a 90% power with a 5% type I error with an allocation ratio of 1:1. We used a 2-sample 2-tailed *t* test for the power analysis.

To conduct the descriptive analysis of quantitative variables, for each variable, we compiled position parameters and dispersion parameters. To describe qualitative variables such as sex or type of practice, we compiled the headcounts and proportions for each modality in the sample.

Comparisons of quantitative variables between groups were performed using a Poisson regression model for counting variables, a linear model for Gaussian variables, or a nonparametric Mann-Whitney-Wilcoxon test for non-Gaussian variables. For comparisons between more than 2 groups, we relied either on ANOVA (for the Gaussian data) or the Kruskal-Wallis test (non-Gaussian data). The correlation between the quantitative variables was measured using the Pearson correlation coefficient or the Spearman correlation coefficient.

A multivariate analysis was performed with all variables. We used a stepwise selection method based on the minimization of the Akaike information criterion. Analyses were performed using R software (version 3.2.2; The R Project for Statistical Computing).

### Ethical Considerations

We obtained ethics committee approval from the Ethical Reflection Group of the Groupe hospitalier Sélestat-Obernai on June 20, 2017, for this study (GH/SO 20062017CY/KR). Participants contacted via email received written study information, whereas those contacted via phone were informed orally. In all cases, oral informed consent was obtained prior to data collection. This approach aligns with French regulatory requirements, specifically a 2016 law (Décret n° 2016-1537 du 16 novembre 2016 relatif aux recherches impliquant la personne humaine [42]) that distinguishes between research involving human subjects and studies that do not fall under this category.

Our study is classified as a noninterventional research not involving human subjects (as defined by Articles L. 1121‐1 and R. 1121‐1 of the French Public Health Code). For such studies, oral consent is considered sufficient, provided that participants are fully informed about the study’s purpose, procedures, and their rights—including the right to withdraw at any time. This approach is consistent with ethical guidelines for medical education research in France, where the emphasis is on ensuring clarity, transparency, and participant autonomy without imposing unnecessary administrative burdens.

All data were anonymized. No compensation was provided to participants.

## Results

### Population

In total, 40 PCPs participated in the study. Half of the participants were women. The mean age of participants was 43 (SD 13) years, and the median age was 37 years (range 31-56.8). The average number of years of experience was 15.1 (minimum 1 to maximum 40; SD 13.6). Half of the participants had more than 10 years of experience. The average STAI-Y score was 37 (SD 8.6; 95% CI 34.2‐39.8), indicating a low level of anxiety. The average PRU score was 17.6 (SD 6.9; 95% CI 15.4‐19.8). Participants rated the scenario’s uncertainty at 3.1 out of 5 (SD 0.9) and its authenticity at 4.4 out of 5 (SD 0.8).

Participants had, on average, 3 final diagnostic hypotheses (SD 0.9; minimum 2 to maximum 5), with an average CCMU score of 2.5 (SD 0.4; minimum 1.5 to maximum 3.7). They requested an average of 9.2 additional investigations and referrals (SD 3.6; minimum 1 to maximum 16), with 34 different proposals. The average cost of additional investigations and referrals per participant was €141.80 (SD 81.6; min 17.5-max 440.5; at the time of the study, the conversion rate of US$ to € was 0.8867). Details are provided in Table 1.

The group with less than 10 years of experience had, on average, fewer regular patients (158 vs 745; *P*<.001) and higher STAI-Y (mean 41.3, SD 6.8 vs mean 32.7, SD 8.2; *P*<.001) and PRU scores (mean 20.7, SD 5.4 vs mean 14.4, SD 6.9; *P*=.002).

Both groups were equivalent in terms of gender, place and type of practice, uncertainty, authenticity of the case, mean CCMU scores, main diagnostic hypothesis’ CCMU score, and average duration of consultations (Table 1).

**Table 1.** Univariate analysis of a group of primary care physicians with less than 10 years of experience vs more than 10 years in a cross-sectional study of experience and additional investigations and referral (AIR) requests in uncertain situations using standardized virtual patient simulations in France in 2017.

	Total (N=40)	<10 years of experience (n=20)	>10 years of experience (n=20)
Age (years), mean (SD)	43.1 (13.8)	31.3 (2.5)	54.9 (9.5)
Women, n (%)	20 (50)	7 (35)	13 (65)
Experience (years), mean (SD)	15.1 (13.6)	3.6 (2.3)	26.5 (9.9)
Practice setting, n (%)			
Alone	26 (65)	14 (70)	12 (60)
Association	14 (35)	6 (30)	8 (40)
Practice location, n (%)			
Urban	25 (62.5)	11 (55)	14 (70)
Semiurban	15 (37.5)	9 (45)	6 (30)
Regular in-office patients, mean (SD)	451 (413)	158 (285.9)	745 (443.9)
Locum PCPs^a^, n (%)	14 (35)	12 (6)	2 (10)
Duration of consultations (minutes), n (%)			
<10	2 (5)	1 (5)	1 (5)
10‐20	32 (80)	16 (80)	16 (80)
20‐30	6 (15)	3 (15)	3 (15)
Uncertainty rating, mean (SD)	3.1 (0.1)	3.3 (0.8)	2.9 (1.0)
Authenticity rating, mean (SD)	4.4 (0.8)	4.5 (0.7)	4.3 (0.9)
STAI-Y^b^ score, mean (SD)	37.0 (8.6)	41.3 (6.8)	32.7 (8.2)
PRU^c^ score, mean (SD)	17.6 (6.9)	20.7 (5.4)	14.4 (6.8)
Number of AIRs, mean (SD)	9.2 (3.6)	10.2 (3.4)	8.1 (3.7)
Price of AIRs (€^d^), mean (SD)	141.8 (81.6)	153.8 (91.4)	129.6 (70.8)
Diagnostic hypotheses, mean (SD)	3.0 (0.9)	3.1 (1.0)	2.9 (0.9)
CCMU^e^ score, mean (SD)	2.5 (0.5)	2.5 (0.5)	2.5 (0.5)

a PCP: primary care physicians

b STAI-Y: State-Trait Anxiety Inventory Form Y.

c PRU: Physician Reaction to Uncertainty.

d At the time of the study, the conversion rate of US$ to € was 0.8867.

e CCMU: classification clinique des malades des urgences.

### Primary End Point

Participants with less than 10 years of professional experience ordered more additional investigations and referrals on average: 10.2 (SD 3.4; 95% CI 8.9‐11.7) vs 8.1 (SD 3.7; 95% CI 6.5‐9.9; *P*=.03). The difference was mostly in laboratory testing, such as C-reactive protein, liver and kidney function tests, and complete blood count. The effect size was 0.603 (95% CI −0.052 to 1.257). There was no effect on costs: €153.8 vs €129.6 (effect size 0.296, 95% CI −0.348 to 0.939; *P*=.23).

In multivariate analysis, there was an association between the number of additional investigations and referrals with age (relative risk [RR] 0.980, 95% CI 0.963‐0.997; adjusted *P*=.02) and mean STAI-Y score (RR 0.984, 95% CI 0.968‐0.999; adjusted *P*=.04) but not with years of experience categorized in 10-year thresholds (*R*^2^=0.308; Table 2).

**Table 2.** Final model of the number of additional investigations and referrals after stepwise backward selection in a cross-sectional study on experience and additional investigations and referrals requests by primary care physicians in uncertain situations using standardized virtual patient simulations in France in 2017.

	Adjusted relative risk (95% CI)		Adjusted *P* value
Experience (>10 years)	1.042 (0.673‐1.611)		.85
Age (years)	0.980 (0.963‐0.997)		.02
Number of regular in-office patients	1.000 (0.999‐1.000)		.15
STAI-Y^a^ score	0.984 (0.968‐0.999)		.04
Sex (men)	0.842 (0.667‐1.064)		.14
Number of hypotheses	1.093 (0.980‐1.218)		.11

a STAI-Y: State-Trait Anxiety Inventory Form Y.

## Discussion

### Principal Findings

Having 10 or more years of experience was not associated with a lower number or costs of additional investigations and referrals ordered in our virtual patient scenario. The group with less than 10 years of experience had significantly higher STAI-Y and PRU scores than the group with more than 10 years of experience, and there was a statistically significant association between STAI-Y score, age, and the number of additional investigations and referrals ordered.

### Comparison With Previous Studies and Interpretation

Unlike many past studies [5,8,14,15], we did not find that greater experience was associated with a decrease in the number of requests for additional investigations and referrals in primary care. There was an association between age and the number of requests in a multivariate analysis. However, neither the difference in the number of requests nor the association with age seemed clinically significant. The difference was an average of 2 additional requests, corresponding to a mean cost difference of less than €25, primarily related to common laboratory tests. Physicians with less than 10 years of experience generated only slightly more diagnostic hypotheses than those with more than 10 years of experience (3.1 vs 2.9). In light of these results, in this virtual study, younger PCPs seem to not significantly rely on additional investigations and referrals to reach a diagnosis in uncertain situations in primary care.

Several factors may explain this discrepancy. First, the study sample may reflect a selection bias, as participation in simulation studies tends to attract clinicians who are more comfortable with digital environments and reflective practice [43]. Second, the use of standardized virtual patients removes key contextual pressures, such as time constraints, patient expectations, and perceived medico-legal risks, all of which are known to influence investigation and referral behaviors in real practice [44]. These contextual pressures might weigh more heavily on less experienced physicians. Third, differences in cognitive load may help explain the findings. According to cognitive load and dual process theories, novices allocate more resources to problem-solving processes, whereas experts rely more on pattern recognition [45-47]. In a virtual environment without external pressures, cognitive load may not lead younger physicians to request more additional investigations and referrals than their senior counterparts. Finally, psychological factors such as tolerance of uncertainty and anxiety—measured using the PRU and STAI-Y—are known to influence investigation behavior (Gerrity et al [48] and Simpkin and Schwartzstein [49]). Our data suggest that while younger PCPs reported higher anxiety and lower tolerance of uncertainty, these traits did not independently predict additional investigations and referrals use once contextual biases were controlled for, indicating that experience alone may be an oversimplified predictor.

In contrast, age and anxiety seemed to be associated with additional investigations and referral requests. The small RR values (0.980 and 0.984) indicate a questionable clinical significance. There were significantly more locum PCPs in the group with less than 10 years of experience (n=12 vs n=2, respectively; *P*<.001). Locum PCPs frequently move from one practice to another. They may therefore lack this feedback loop, which is essential for enhancing diagnostic reasoning and understanding the typical clinical progression of common conditions [50]. This might be part of the explanation of why less experienced PCPs requested more additional investigations and referrals and had significantly higher PRU and STAI-Y scores. Hillen et al [51] conceptualized uncertainty tolerance (UT) as “the set of negative and positive psychological responses—cognitive, emotional, and behavioral—provoked by the conscious awareness of ignorance about particular aspects of the world”. UT could lead to decision deferral as part of a negative behavioral response to uncertainty [51]. This could help explain a higher use of additional investigations and referrals in a more anxious group. The perception of uncertainty could also lead to negative emotional responses, such as worry or fear [51]. Such responses may be part of defensive medical practice [44]. Research shows that physicians sometimes order additional investigations and referrals to reassure patients and themselves [5,20,21,26], but the effect is small on patients [52]. Although such orders may reassure physicians, it would be better to develop interventions aimed at reducing physicians’ anxiety directly rather than accept orders for additional investigations and referrals made simply for reassurance. From a medical education perspective, an effort should be made to improve the practices of primary care residents’ supervisors by offering specific feedback on experience aimed at building up positive responses of UT [14,49,51]. This may promote the optimal use of additional investigations and referrals in primary care by future clinicians.

Many international guidelines insist on the necessity to rationally use additional investigations and referrals after a period of watchful waiting in the absence of red flags in situations such as fatigue or unexplained symptoms [53-56]. In the present case of dyspnea that had progressed for several days in a man aged 69 years, prompt additional investigations and referrals were justified. Participants’ requests were mostly adequate considering the 8 additional investigations and referrals to order in the pregraduated national learning objectives for acute dyspnea: complete blood count, blood electrolyte panel, blood glucose, brain natriuretic peptide or N-terminal prohormone of brain natriuretic peptide, D-dimers, electrocardiography, chest X-ray, and arterial blood gas analysis [57]. Our data suggest that progress could still be made on the rational use of additional investigations and referrals. Interventions such as the “five-step program for diagnostic test addiction” [58] could be used in a primary care setting: decide what diagnosis you are investigating, determine the pretest probability of the condition in question, decide whether to rule the condition in or out, decide what you will do if the test result is positive or negative, and ask whether ordering this test could hurt the patient. It would be interesting to explore the relationship between requests for additional investigations and referrals and clinical experience in scenarios where an experienced physician might choose not to rely on such requests. Such situations could provide valuable insights into how experience influences decision-making and the perceived necessity of additional tests, particularly in cases where clinical confidence may outweigh diagnostic uncertainty.

From a clinical reasoning perspective, experience is conducive to the optimization of knowledge organization in the form of clinical scripts among PCPs (clinical scripts) [4,59]. We hypothesize that with more efficient clinical scripts, PCPs could better identify situations in which to order additional investigations and referrals, when to refrain from ordering them, and when to adopt a watchful, waiting approach [56]. In contrast, Watson et al [26] showed that PCPs developed their own mental shortcuts, including blood tests. Teaching clinical reasoning could incorporate practical exercises based on clinical cases that require decisions about whether to request additional investigations and referrals in situations of uncertainty [60].

The way clinical reasoning is supervised may also positively influence students’ anxiety and tolerance of uncertainty. For instance, the SNAPPS (summarize, narrow, analyze, probe, plan, select) model has been shown to encourage learners to voice more diagnostic uncertainty during supervisory encounters [61-63]. This approach explicitly targets the inherent diagnostic uncertainty of clinical care and may therefore help improve the appropriateness of requests for additional investigations and referrals among future physicians.

### Limitations and Future Directions

The authenticity of the case is limited by the extent of the software’s possibilities and current technical constraints. The software cannot capture certain types of information clinicians use in their reasoning, such as smells, and it is difficult for participants to fully detach from their usual practice context to engage with the simulated consultation environment [4]. The STAI-Y score only included the measurement of state-anxiety (not trait-anxiety). The impact of trait anxiety on additional investigations and referrals requests is not known. Moreover, the requests for additional investigations and referrals were self-reported and may not correctly predict real-life behavior. Experience was only defined by years of practice, although previous research has shown that the correlation between years of experience and clinical performance in medicine is often weak [32]. Qualitative insights on the participants’ expertise defined by peers rather than the number of years of experience could have enriched our comprehension of the topic. The snowball sampling method allowed us to respect the stratification of participant recruitment, ensuring that the sample aligned with the intended population characteristics. However, this method may have introduced some bias into our results, as it could increase the likelihood of finding associations specific to this population that may not exist in the general population [64]. Given that the target population was not particularly difficult to reach, future studies might consider using a larger sample with random sampling method to enhance generalizability and minimize potential biases. We chose to focus on only a portion of the potential determinants of additional investigations and referrals in our data collection for feasibility reasons.

### Conclusions

Less experienced PCPs do not appear to overly rely on additional investigations and referrals in situations of uncertainty, suggesting that clinical reasoning remains a well-preserved skill among the younger generation of physicians. However, these findings should be interpreted with caution, as they may not fully account for the nuances in decision-making across varying levels of experience. Future research could explore interventions targeting physicians’ anxiety and tolerance of uncertainty as potential factors influencing additional investigations and referral requests. In practical terms, 2 avenues could be explored: specific training for supervisors to help them address UT in the feedback they provide to trainees and the use of virtual consultations as a complement to traditional training, with particular attention to this aspect. For instance, using methods such as SNAPPS could improve additional investigations and referral requests among future physicians by explicitly addressing clinical uncertainty. Additionally, it would be valuable to investigate scenarios where experienced physicians might consciously avoid additional investigations and referrals, providing further insights into the interplay between experience and test-ordering behavior in primary care.

## Supplementary material

10.2196/71717 Checklist 1 STROBE checklist.

## Abbreviations

PCP: primary care physician
PRU: Physician Reaction to Uncertainty
RR: relative risk
SNAPPS: summarize, narrow, analyze, probe, plan, select
STAI-Y: State-Trait Anxiety Inventory Form Y
UT: uncertainty tolerance
