# Correction: Additional Investigations and Referrals by Primary Care Physicians in Uncertain Clinical Situations: Cross-Sectional Study Using Virtual Patient–Based Scenarios

**DOI:** 10.2196/98838

**Published:** 2026-08-07

**Authors:** Mathieu Lorenzo, Thierry Pelaccia, Emmanuel Triby, Yunus Can, Romain Krider, Hubert Maisonneuve, Nicolas Fernandez

**Affiliations:** 1 Department of General Medicine and Territorial Training, Faculty of Medicine, Midwifery, and Health Sciences, University of Strasbourg, 4 rue Kirschleger, Strasbourg, 67085, France, 33 03 68 85 34 55; 2 Center for Training and Research in Health Sciences Education (CFRPS), Faculty of Medicine, Midwifery, and Health Sciences, University of Strasbourg, Strasbourg, Grand Est, France; 3 Faculty of Education and Lifelong Learning (ESPE), University of Strasbourg, Strasbourg, France; 4 University Institute for Primary Care, Faculty of Medicine, University of Geneva, Geneva, Switzerland; 5 Center for Applied Pedagogy in Health Sciences (CPASS), University of Montreal, Montréal, QC, Canada

In “Additional Investigations and Referrals by Primary Care Physicians in Uncertain Clinical Situations: Cross-Sectional Study Using Virtual Patient–Based Scenarios” [1], the authors noted three corrections.

In Table 1, where it says:

**Table 1.** 

	Total (N=40)	<10 years of experience (n=20)	>10 years of experience (n=20)
Number of AIRs, mean (SD)	10.3 (3.7)	11.5 (3.5)	9.2 (3.7)

The data are corrected as follows (presented here as Table 2):

**Table 2.** 

	Total (N=40)	<10 years of experience (n=20)	>10 years of experience (n=20)
Number of AIRs, mean (SD)	9.2 (3.6)	10.2 (3.4)	8.1 (3.7)

In the results section, where it says:

They requested an average of 10.3 additional investigations and referrals (SD 3.6; minimum 1 to maximum 16), with 34 different proposals.

The text has been replaced with:

They requested an average of 9.2 additional investigations and referrals (SD 3.6; minimum 1 to maximum 16), with 34 different proposals.

In the results section, where it says:

Participants with less than 10 years of professional experience ordered fewer additional investigations and referrals on average: 10.2 (SD 3.4; 95% CI 8.9‐11.7) vs 8.1 (SD 3.7; 95% CI 6.5‐9.9; *P*=.02).

The text has been replaced with:

Participants with less than 10 years of professional experience ordered more additional investigations and referrals on average: 10.2 (SD 3.4; 95% CI 8.9‐11.7) vs 8.1 (SD 3.7; 95% CI 6.5‐9.9; *P*=.03).

These corrections will appear in the online version of the paper on the JMIR Publications website together with the publication of this correction notice. Because this was made after submission to PubMed, PubMed Central, and other full-text repositories, the corrected article has also been resubmitted to those repositories.
